# HIV Testing, Linkage to HIV Medical Care, and Interviews for Partner Services Among Black Men Who Have Sex with Men — Non–Health Care Facilities, 20 Southern U.S. Jurisdictions, 2016

**DOI:** 10.15585/mmwr.mm6728a3

**Published:** 2018-07-20

**Authors:** Mariette Marano, Renee Stein, Wei Song, Deesha Patel, Nicole Taylor-Aidoo, Songli Xu, Lamont Scales

**Affiliations:** ^1^Division of HIV/AIDS Prevention, National Center for HIV/AIDS, Viral Hepatitis, STD, and TB Prevention, CDC; ^2^Keymind, a division of Axiom Resource Management, Inc., Falls Church, Virginia.

Identifying HIV-infected persons who are unaware of their human immunodeficiency virus (HIV) infection status, linking them to care, and reducing health disparities are important national HIV prevention goals (*1*). Gay, bisexual, and other men who have sex with men (collectively referred to as MSM) accounted for 70% of HIV infection diagnoses in the United States in 2016, despite representing only 2% of the population (*2*,*3*). African American or black (black) MSM accounted for 38% of all new diagnoses of HIV infection among MSM (*2*). Nearly two thirds (63%) of all U.S. black MSM with diagnosed HIV infection reside in the southern United States (*2*), making targeted HIV prevention activities for black MSM in this region critical. Analysis of CDC-funded HIV testing data for black MSM submitted by 20 health departments in the southern United States in 2016 revealed that although black MSM received 6% of the HIV tests provided, they accounted for 36% of the new diagnoses in non–health care facilities. Among those who received new diagnoses, 67% were linked to HIV medical care within 90 days of diagnosis, which is below the 2020 national goal of linking at least 85% of persons with newly diagnosed HIV infection to care within 30 days (*1*). Black MSM in the southern United States are the group most affected by HIV, but only a small percentage of CDC tests in the southern United States are provided to this group. Increasing awareness of HIV status through HIV testing, especially among black MSM in the southern United States, is essential for reducing the risk for transmission and addressing disparities. HIV testing programs in the southern United States can reach more black MSM by conducting targeted risk-based testing in non–health care settings and by routine screening in agencies that also provide health care services to black MSM.

In 2016, CDC funded 20 health departments and 24 community-based organizations (CBOs) to provide HIV testing and related services in the southern United States. Health departments and CBOs submitted deidentified program data about services provided through a secure, online CDC-supported system. Data from 2016, analyzed for this report, include the number of CDC-funded HIV tests,* new HIV-positive diagnoses, information on linkage of persons with newly or previously identified HIV infection to medical care within 90 days,^†^ and interviews for partner services.^§^ Analyses were restricted to HIV tests provided in the 20 southern U.S. jurisdictions^¶^ in non–health care facilities,** to persons who reported their sex at birth and current gender identity as male, reported sex with a male in the preceding 12 months, and their age as ≥13 years. Non–health care facilities routinely collect HIV-related risk information from all clients, whereas health care facilities are only required to collect HIV risk information from HIV-positive clients. Data were stratified by the following characteristics: age group, first-time tested, and urbanicity. Urbanicity was based on the 2013 Urban-Rural Classification Scheme for Counties of the National Center for Health Statistics; for this analysis, the categories included metropolitan (population of ≥1,000,000), urban (50,000–999,999), or rural (<50,000). Multivariate binomial regression was used to assess the association between demographic characteristics and newly diagnosed HIV infections, linkage to HIV medical care, and interviews for partner services.

Among the 374,871 CDC-funded HIV tests provided in non–health care facilities in the 20 southern jurisdictions in 2016, a total of 22,183 (6%) were provided to black MSM, who accounted for 828 (36%) of 2,304 new diagnoses of HIV infection among all persons tested in non–health care facilities in these jurisdictions.^††^ Among black MSM in the jurisdictions, the highest percentages of tests were provided to men aged 25–34 years (43%), living in metropolitan areas (75%), and who had been tested previously (81%) (Table 1). Overall, 1,471 black MSM had positive tests for HIV infection in 2016; among these, 828 (56%) received a new diagnosis (Table 2) and 643 (44%) had previously received a diagnosis of HIV infection (Table 3). Among black MSM, new diagnoses of HIV infection were highest in persons aged 20–24 years (4.8%) followed by those aged 13–19 (4.1%) and 25–34 (4.0%) years. Compared with black MSM aged 25–34 years, those aged ≥35 years were less likely to receive a new diagnosis (adjusted prevalence ratio [aPR] 35–44 years = 0.56; aPR 45–54 years = 0.46; and aPR ≥55 years = 0.34). Compared with black MSM who had not been tested before, those who reported a previous HIV test were less likely to receive a new HIV diagnosis (aPR = 0.73). Overall, 608 (73%) new diagnoses of HIV infection were in persons tested in a metropolitan area. Compared with tests performed in metropolitan jurisdictions, tests performed in urban jurisdictions were more likely to yield new diagnoses, and tests performed in rural jurisdictions were less likely to yield new diagnoses (aPR = 1.23 and 0.48, respectively) (Table 1).

**TABLE 1:** HIV tests and newly diagnosed HIV infections among black gay, bisexual, and other men who have sex with men (MSM) in non–health care facilities, by selected characteristics — 20 southern U.S. jurisdictions, 2016

Characteristic	Total no. of HIV tests*	HIV tests among black MSM		Total no. of newly diagnosed HIV infections^†^	Newly diagnosed HIV infections among black MSM			
		No. (%)	(Row %)		No. (%)	(Row %)	% positive	aPR (95%CI)
Total	374,871	22,183 (100.0)	5.9	2,304	828 (100.0)	35.9	3.7	—
**Age group (yrs)** ^§^								
13–19	30,815	1,404 (6.3)	4.6	99	58 (7.0)	58.6	4.1	0.97 (0.73–1.29)
20–24	81,589	6,060 (27.3)	7.4	571	289 (34.9)	50.6	4.8	1.16 (0.99–1.36)
25–34	121,731	9,508 (42.9)	7.8	921	378 (45.7)	41.0	4.0	Referent
35–44	61,739	2,645 (11.9)	4.3	353	63 (7.6)	17.9	2.4	0.56 (0.43–0.75)^¶^
45–54	44,662	1,556 (7.0)	3.5	238	27 (3.3)	11.3	1.7	0.46 (0.31–0.68)^¶^
≥55	32,434	949 (4.3)	2.9	113	12 (1.5)	10.6	1.3	0.34 (0.19–0.60)^¶^
**First-time tested** ^§^								
Yes	79,967	3,630 (16.4)	4.5	513	163 (19.7)	31.8	4.5	Referent
No	224,395	17,848 (80.5)	8.0	1,690	635 (76.7)	37.6	3.6	0.73 (0.61–0.87)^¶^
**Urbanicity** ^§^								
Metropolitan	235,666	16,559 (74.7)	7.0	1,669	608 (73.4)	36.4	3.7	Referent
Urban	89,010	4,076 (18.4)	4.6	531	188 (22.7)	35.4	4.6	1.23 (1.05–1.45)**
Rural	41,643	587 (2.7)	1.4	32	12 (1.5)	37.5	2.0	0.48 (0.27–0.86)**

**Abbreviations:** aPR = adjusted prevalence ratio; CI = confidence interval; HIV = human immunodeficiency virus.

* HIV tests were defined as tests for which a result (i.e., positive or negative) was known. Analyses excluded discordant and indeterminate results.

^†^ Included are persons who tested HIV-positive and did not report a previous positive test result, calculated using HIV surveillance verification (if available) or a person’s self-reported previous HIV status.

^§^ Missing/invalid data were excluded. In the column “HIV tests among black MSM,” 61 (0.3%) records were excluded from the age group category, 705 (3.2%) from the first-time tested category, and 961 (4.3%) from the urbanicity category. In the column “Total no. of newly diagnosed HIV infections,” nine (0.4%) records were excluded from the age group category, 101 (4.4%) from the first-time tested category, and 72 (3.1%) from the urbanicity category. In the section “Newly diagnosed HIV infections among black MSM,” one (0.1%) record was excluded from the age group category, 30 (3.6%) from the first-time tested category, and 20 (2.4%) from the urbanicity category.

^¶^ p-value <0.001.

** p-value <0.05.

**TABLE 2:** Linkage to HIV medical care and interview for partner services among HIV-positive black gay, bisexual, and other men who have sex with men (MSM) with newly diagnosed HIV infection in non–health care facilities, by selected characteristics — 20 southern U.S. jurisdictions, 2016

Characteristic	No. of newly diagnosed HIV infections*	Linked to HIV medical care within 90 days of diagnosis^†^			Interviewed for HIV partner services^§^		
		No. (row %)	Missing no. (%)	aPR (95% CI)	No. (row %)	aPR (95% CI)	Missing, No. (%)
**Total**	**828**	**552 (66.67)**	**180 (21.74)**	**—**	**451 (54.5)**	**—**	**174 (21.0)**
**Age group (yrs)**							
13–19	58	43 (74.1)	8 (13.8)	1.08 (0.91–1.29)	35 (60.3)	1.26 (0.99–1.60)	8 (13.8)
20–24	289	187 (64.7)	65 (22.5)	0.94 (0.84–1.05)	177 (61.3)	1.23 (1.06–1.42)^¶^	55 (19.0)
25–34	378	262 (69.3)	81 (21.4)	Referent	189 (50.0)	Referent	87 (23.0)
35–44	63	38 (60.3)	14 (22.2)	0.90 (0.72–1.11)	34 (54.0)	1.08 (0.82–1.42)	13 (20.6)
45–54	27	16 (59.3)	8 (29.6)	0.85 (0.62–1.17)	13 (48.2)	1.00 (0.67–1.50)	5 (18.5)
≥55	12	6 (50.0)	4 (33.3)	0.72 (0.41–1.27)	3 (25.0)	0.52 (0.20–1.41)	6 (50.0)
First-time tested**							
Yes	163	105 (64.4)	36 (22.1)	Referent	86 (52.8)	Referent	48 (29.5)
No	635	423 (66.6)	139 (21.9)	1.04 (0.92–1.18)	342 (53.9)	1.07 (0.91–1.25)	121 (19.0)
Urbanicity**							
Metropolitan	608	413 (67.9)	138 (22.7)	Referent	323 (53.1)	Referent	136 (22.4)
Urban	188	123 (65.4)	38 (20.2)	0.97 (0.86–1.09)	105 (55.9)	1.05 (0.91–1.22)	37 (19.7)
Rural	12	6 (50.0)	1 (8.3)	0.75 (0.43–1.33)	10 (83.3)	1.44 (1.10–1.90)^¶^	0 (0.0)

**Abbreviations:** aPR = adjusted prevalence ratio; CI = confidence interval; HIV = human immunodeficiency virus.

* Included persons who tested HIV-positive during the current test and were not found to be previously reported in the health department jurisdiction’s HIV surveillance system or who self-reported not having a previous HIV-positive test result if surveillance system verification was not available.

^†^ Linkage to HIV medical care within 90 days means confirmation that persons attended their first HIV medical care appointment within 90 days of their HIV test date.

^§^ Partner services is a process through which HIV-infected persons are interviewed to elicit information about their partners, who can then be confidentially notified of their possible exposure or potential risk and offered services that can protect the health of partners and prevent HIV transmission to others.

^¶^ p-value <0.01.

** Missing/invalid data were excluded. In the column “No. of newly diagnosed HIV infections,” one (0.1%) record was excluded from the age group category, 30 (3.6%) from the first-time tested category, and 10 (1.2%) from the urbanicity category. In the section “Linked to HIV medical care within 90 days of diagnosis,” 24 (4.3%) records were excluded from first-time tested and 10 (1.8%) from the urbanicity category. In the section “Interviewed for HIV partner services,” 23 (5.1%) records were excluded from first-time tested and 13 (2.9%) from urbanicity.

**TABLE 3:** Linkage to HIV medical care among HIV-positive black gay, bisexual, and other men who have sex with men (MSM) with a previous diagnosis of HIV infection in non–health care facilities — 20 southern U.S. jurisdictions, 2016

Characteristic	Previously diagnosed HIV infection*	Previously diagnosed HIV-positive black MSM linked to HIV medical care^†^		
	No.	No. (%)	aPR (95% CI)	Missing, No. (%)
**Total**	**643**	**374 (58.2)**	**—**	**116 (18.0)**
**Age group (yrs)** ^§^				
13–19	25	19 (76.0)	1.19 (0.90–1.57)	3 (12.0)
20–24	149	86 (57.7)	0.96 (0.82–1.14)	27 (18.1)
25–34	309	189 (61.2)	Referent	57 (18.5)
35–44	81	47 (58.0)	0.94 (0.76–1.18)	13 (16.1)
45–54	51	21 (41.2)	0.70 (0.49–1.00)^¶^	11 (21.6)
≥55	25	11 (44.0)	0.73 (0.44–1.20)	4 (16.0)
**First-time tested** ^§^				
Yes	75	52 (69.3)	Referent	4 (5.3)
No	551	311 (56.4)	1.04 (0.75–1.45)	110 (20.0)
**Urbanicity** ^§^				
Metropolitan	443	232 (52.4)	Referent	94 (21.2)
Urban	112	78 (69.6)	1.36 (1.16–1.58)**	20 (17.9)
Rural	48	36 (75.0)	1.38 (0.94–2.04)	0 (0.0)

**Abbreviations:** aPR = adjusted prevalence ratio; CI = confidence interval; HIV = human immunodeficiency virus.

* Previously diagnosed HIV infections included persons who tested HIV-positive during the current test and were found to be previously reported in the health department’s HIV surveillance system or who self-reported having a previous HIV-positive test result if the surveillance system verification was not available.

^†^ Linkage to HIV medical care within 90 days means confirmation that persons attended their first HIV medical care appointment within 90 days of their HIV test date.

^§^ Missing/invalid data were excluded. In the section “Previously diagnosed HIV-positive black MSM who are linked to HIV medical care,” one (0.3%) record was excluded from the age group category, 11 (2.9%) from first-time tester, and 28 (7.5%) from urbanicity.

^¶^ p-value <0.05.

** p-value <0.001.

In this analysis, among the 828 black MSM in the southern U.S. jurisdictions with newly diagnosed HIV infection, 552 (67%) were linked to HIV medical care within 90 days of diagnosis, and 451 (55%) were interviewed for partner services. The percentage of black MSM with newly diagnosed HIV infection who were interviewed for partner services was higher among persons aged 20–24 years (61%) than among those aged 25–34 years (50%) (aPR = 1.23). In addition, the percentage of black MSM with newly diagnosed HIV infection who were interviewed for partner services was higher in rural jurisdictions (83%) than in metropolitan jurisdictions (53%) (aPR = 1.44) (Table 2). Black MSM with newly diagnosed HIV infections were significantly more likely to be linked to HIV medical care (odds ratio = 1.44, p = 0.0008) than were those with a previously diagnosed infection.

Among the 643 black MSM in the southern U.S. jurisdictions with a previously diagnosed HIV infection, 374 (58%) were linked to HIV medical care within 90 days of the test date. The adjusted prevalence ratio of being linked to HIV medical care within 90 days was higher for those living in urban areas (70%) than for those living in metropolitan areas (52%) (aPR = 1.36) (Table 3).

## Discussion

HIV testing and prompt linkage to and retention in HIV medical care are essential to achieve viral suppression among those HIV-positive persons unaware of their infection or aware but not in care. (*4,5*). The findings from this study highlight the value of CDC’s HIV testing program for reaching black MSM in the southern United States who are at highest risk for acquiring or transmitting HIV infection. Among black MSM in 20 southern U.S. jurisdictions, the percentage of HIV-positive results was highest among men aged <35 years (4.3%), highlighting the critical importance of prioritizing this population. However, given that black MSM accounted for only 6% of HIV tests but 36% of new diagnoses, efforts to increase HIV testing of black MSM in non–health care facilities in the southern United States are needed. Approximately half (44%) of the positive HIV test results were among black MSM with previously diagnosed infections, underscoring the need to prioritize testing among black MSM who have never been tested for HIV.

Approximately two thirds (67%) of HIV-positive black MSM in these southern jurisdictions with newly diagnosed infection, and 58% with previously diagnosed infection, were linked to HIV medical care, both short of the national goal of 85% (*1*). Black MSM with previously diagnosed HIV infection might have been linked to HIV medical care upon initial diagnosis and subsequently fallen out of care. Their return to HIV testing might indicate willingness to be linked or reengaged in care; however, these men with previously diagnosed infections might face more obstacles to accessing care than would someone with a new HIV diagnosis, particularly if they are linked back into the same health system that failed them initially (*6*). For black MSM in the southern United States, racism, lower educational levels, stigma, income inequality, and lack of access to health care are barriers to testing, linkage, and retention in HIV prevention and treatment services (*7*,*8*). In addition, some persons living with HIV infection in the rural southeastern United States might have to travel >50 miles to receive HIV care (*9*).

The findings in this report are subject to at least four limitations. First, findings describe CDC-funded HIV tests only and are not generalizable to HIV testing rates among all black MSM in the southern United States or in the entire United States. Second, linkage data include records with missing or invalid data in the denominator, and therefore probably underestimate the percentage of persons linked to care. Third, when surveillance data are unavailable to verify prior HIV status, the number of new positive results might be overestimated if clients inaccurately report their HIV testing history. Finally, findings describe only tests provided in non–health care facilities because these facilities collect HIV-related risk information from all clients, whereas health care facilities only routinely collect HIV risk information from HIV-positive clients.

Increasing HIV testing among black MSM in the southern United States is essential for reducing HIV infection in this disproportionately affected population. However, the efficiency and effectiveness of this approach is contingent upon reaching MSM who are living with undiagnosed HIV infection. HIV testing programs in the southern United States can be designed to reach more black MSM who are unaware of their HIV status either by conducting targeted risk-based testing in non–health care settings (e.g., outreach) or routine screening in agencies that also provide health care services to black MSM. HIV testing programs in the southern United States also need to improve linkage to HIV medical care among HIV-positive black MSM who are not in care.

**Summary**

**What is already known about this topic?**

Black men who have sex with men (MSM) are disproportionately affected by human immunodeficiency virus (HIV) infection, accounting for 38% of all new HIV diagnoses among MSM in the United States in 2016.

**What is added by this report?**

Analysis of CDC-funded HIV testing for black MSM in 20 southern U.S. jurisdictions in 2016 revealed that black MSM received 6% of the HIV tests provided and accounted for 36% of the new HIV diagnoses in non–health care facilities.

**What are the implications for public health practice?**

HIV testing programs in the southern United States can be designed to reach more black MSM by conducting targeted risk-based testing in non–health care settings and by routine screening in agencies that also provide health care services to black MSM.

## Footnotes

* An HIV test is defined as the performance of one or more HIV tests to determine a person’s HIV infection status. A person might be tested once (e.g., one rapid test or one conventional test) or multiple times (e.g., one rapid test followed by one conventional test to confirm a preliminary HIV-positive test result).

† Linkage to HIV medical care within 90 days means confirmation that persons attended their first HIV medical care appointment within 90 days of their HIV test date.

§ Partner services is a process through which HIV-infected persons are interviewed to elicit information about their partners, who can then be confidentially notified of their possible exposure or potential risk and offered services that can protect the health of partners and prevent HIV transmission to others.

¶ Southern U.S. jurisdictions included the following 16 states: Alabama, Arkansas, Delaware, Florida, Georgia, Kentucky, Louisiana, Maryland, Mississippi, North Carolina, Oklahoma, South Carolina, Tennessee, Texas, Virginia, and West Virginia. Also included were the following four jurisdictions: Atlanta, Baltimore, District of Columbia, and Houston.

** Non–health care facilities are settings where HIV testing is performed using a targeted testing strategy rather than a routine screening strategy. Examples of non–health care facilities include HIV testing sites (such as an HIV testing event or HIV testing mobile van) and community settings (such as an AIDS service organization).

†† Among the 11,710 newly diagnosed HIV-positive persons identified by CDC-funded HIV tests provided in health care and non–health care facilities in 2016, 2,327 (20%) were white, 5,676 (48%) were black, 2,943 (25%) were Hispanic or Latino, 229 (2%) were Asian, 57 (0.5%) were American Indian or Alaska Native, 18 (0.2%) were Native Hawaiian or Pacific Islander, and 122 (1%) were multiracial.

## References

[1] Office of National AIDS Policy. National HIV/AIDS strategy for the United States: updated to 2020. Washington, DC: Office of National AIDS Policy; 2015. https://obamawhitehouse.archives.gov/sites/default/files/docs/national_hiv_aids_strategy_update_2020.pdf

[2] CDC. HIV surveillance report: diagnoses of HIV infection in the United States and dependent areas, 2016. Atlanta, GA: US Department of Health and Human Services, CDC; 2017. https://www.cdc.gov/hiv/pdf/library/reports/surveillance/cdc-hiv-surveillance-report-2016-vol-28.pdf

[3] Grey JA, Bernstein KT, Sullivan PS, Estimating the population sizes of men who have sex with men in the US states and counties using data from the American community survey. JMIR Public Health Surveill 2016;2:e14. 10.2196/publichealth.5365 27227149 PMC4873305

[4] Cohen MS, Chen YQ, McCauley M, ; HPTN 052 Study Team. Prevention of HIV-1 infection with early antiretroviral therapy. N Engl J Med 2011;365:493–505. 10.1056/NEJMoa1105243 21767103 PMC3200068

[5] Lundgren JD, Babiker AG, Gordin F, ; INSIGHT START Study Group. Initiation of antiretroviral therapy in early asymptomatic HIV infection. N Engl J Med 2015;373:795–807. 10.1056/NEJMoa1506816 26192873 PMC4569751

[6] Seth P, Wang G, Belcher L. Previously diagnosed HIV-positive persons: the role of Centers for Disease Control and Prevention–funded HIV prevention programs in addressing their needs. Sex Transm Dis 2018;45:377–81. 10.1097/OLQ.0000000000000766 29465676 PMC11289746

[7] Buot M-LG, Docena JP, Ratemo BK, Beyond race and place: distal sociological determinants of HIV disparities. PLoS One 2014;9:e91711. 10.1371/journal.pone.0091711 24743728 PMC3990614

[8] Piper K, Enah C, Daniel M. Black southern rural adolescents’ HIV stigma, denial, and misconceptions and implications for HIV prevention. J Psychosoc Nurs Ment Health Serv 2014;52:50–6. 10.3928/02793695-20140210-01 24530218

[9] Lopes BLW, Eron JJ Jr, Mugavero MJ, Miller WC, Napravnik S. HIV care initiation delay among rural residents in the Southeastern United States, 1996 to 2012. J Acquir Immune Defic Syndr 2017;76:171–6. 10.1097/QAI.0000000000001483 28639994 PMC5850947

